# Mental health treatment and access for emerging adults in Canada: a systematic review

**DOI:** 10.3389/fpubh.2023.1088999

**Published:** 2023-07-10

**Authors:** Jonah Kynan Murray, Sarah Knudson

**Affiliations:** ^1^Department of Psychology & Health Studies, University of Saskatchewan, Saskatoon, SK, Canada; ^2^St. Thomas More College, Saskatoon, SK, Canada

**Keywords:** emerging adulthood, mental health, healthcare, Canada, health education

## Abstract

**Introduction:**

Research into the mental healthcare of emerging adults (18–25) in Canada has been limited, despite this developmental period being widely considered a vulnerable time of life. As such, we aimed to identify the greatest barriers emerging adults faced in accessing mental healthcare in Canada, particularly in relation to the Canadian healthcare system which operates on a universal funding model but is challenged by funding shortfalls and a complex relationship to the provinces.

**Methods:**

We systematically examined 28 pieces of literature, including academic and technical literature and publications from government organizations, focused on emerging adults and the Canadian mental healthcare system.

**Results:**

Findings demonstrated that stigma, a lack of mental health knowledge, cost, and interpersonal factors (e.g., one’s parental, peer, and romantic supports demonstrating negative views toward mental healthcare may deter treatment; emerging adults demonstrating concerns that accessing mental healthcare may lead to peer rejection) acted as barriers to help-seeking in emerging adults. Additionally, a lack of national institutional cohesion and a lack of policy pertaining to emerging adult healthcare acted as barriers to adequate mental healthcare in this demographic.

**Discussion:**

Improving mental health education early in life shows promise at reducing many of the barriers emerging adults face in accessing mental healthcare. Further, policies directed at ensuring a cohesive national mental health system, as well as policies directly designed to care for emerging adult mental health needs, could act as the next steps toward ensuring an accessible and effective Canadian mental healthcare system that can serve as a model for other nations.

## 1. Introduction

Research into how emerging adults, typically operationalized as youth 18–25 years of age (1–3), understand and experience mental health and its effects has been relatively sparse, as studies into youth have been primarily focused on depression in teenagers (4). This is surprising considering how emerging adulthood has been consistently characterized as a particularly susceptible period of life for the development of mental health disorders (4–8). Emerging adults begin to face new and stressful educational and work situations at this juncture, and begin to take on social roles in which they are inexperienced (4, 6, 7). Within this literature, research into emerging adults’ understanding and experiences of mental health effects has been particularly limited regarding their capabilities in accessing mental health resources. This is especially pronounced in the Canadian context, where accessing mental health resources can be challenging for emerging adults as mental healthcare policies are not uniform throughout the country and are implemented independently by provincial and territorial governments (9).

Taken together, these factors suggest that Canada’s emerging adults are particularly likely to face barriers in seeking mental health treatment. As such, in order to best determine how to support emerging adults’ mental health, it is of vital importance to query the state of mental healthcare in Canada with regard to emerging adults. It is also important to assess this age group’s mental health literacy–as reported in existing academic and gray literature–which encompasses knowledge and recognition of mental health disorders (6) and access to mental health resources for emerging adults. We seek to better understand existing barriers by conducting a systematic review of relevant literature after first framing the issue within its broader academic and national context. Ultimately, our aim is to identify shortcomings in the Canadian system that may assist other nations in recognizing and addressing their respective challenges with young adults’ mental healthcare.

We are primarily interested in the age range of 18–26 given its aforementioned risks and timing as a critical developmental juncture, and we utilize Arnett’s (1) theory of emerging adulthood as a heuristic tool for understanding the importance of mental healthcare in this developmental period. Arnett’s (1) theory offers insights into the unique developmental challenges associated with this time of life, along with its potential risk factors that may correlate with barriers to mental health resources. Ultimately, the findings of this review are aimed at directing future efforts to adapt and refine Canadian healthcare policies to better serve the mental healthcare needs of emerging adults. By extension, it aims to provide a roadmap whereby other nations can identify and address their unique mental healthcare hurdles for young adults.

### 1.1. Approach to identifying structural shortcomings

The primary purpose of this review is to identify where the Canadian healthcare system is lacking in relation to the education and treatment of emerging adults’ mental health issues at both the federal and provincial levels. First, for context, this paper will present a general literature review pertaining to mental health determinants and barriers for emerging adults, as well as an overview of Canadian healthcare systems and their existing shortcomings. We follow this up with a focused review of literature that looks simultaneously at Canadian mental healthcare and emerging adults. Finally, we present a discussion of findings that considers the research problem within an international context and offers ideas for structural and cultural changes to addressing emerging adults’ mental healthcare.

The focus of this first portion of our review centers on understanding emerging adulthood as it relates to mental healthcare, and becoming familiar with federal and provincial policies relating to mental health treatment; thus, it is guided by the following overarching question: What are the most common barriers that emerging adults face in seeking mental health treatment? Our central question precedes a number of sub-questions: (1) Where and how–in terms of jurisdictional levels, policies, and administrative structures–does the Canadian healthcare system lack vis-à-vis caring for the mental health of emerging adults? And (2) What steps can we take to improve mental healthcare among emerging adults? (3) What internationally applicable findings can be drawn from this systematic review?

### 1.2. Emerging adulthood: hallmarks and mental healthcare considerations

Developmental psychologist Jeffrey J. Arnett first proposed the term emerging adulthood and theorized about the nature and importance of this developmental stage (2000). Here, we use it as a heuristic tool for understanding our focal age demographic (18–25 years) as it offers insights into the unique developmental and mental health risk factors at this time of life. Arnett defined emerging adulthood as:

A time of life when many different directions remain possible, when little about the future has been decided for certain, when the scope of independent exploration of life’s possibilities is greater for most people than it will be at any other period of the life course (1).

This theory originated by analyzing the works of other developmental theorists (i.e., Erikson, 1950, 1968; Keniston, 1971; Levinson, 1978) who also identified a unique developmental period around this time of the life course.

Arnett (1), however, contends that since the time of the earlier developmental theorizations, the nature of young adulthood has changed considerably; notably, there has been a general delay in the timing of marriage and parenthood, and the only role stability in this developmental period (meaning the most recurrent characteristics) is that there is *very little role stability*, as emerging adults engage in a period of experimentation and exploration in terms of education, living situations, and general autonomy. Subjectively, Arnett notes that emerging adults do not see themselves as adolescents nor entirely as adults, and, at least in modern westernized cultures, it is not until their late twenties and early thirties that Arnett indicates that the majority of people feel they have reached adulthood. Arnett determined that the qualities that indicate that a person sees themself as having reached adulthood are: accepting responsibility for one’s self; making independent decisions; and becoming financially independent. As such, a sense of self-sufficiency seems to indicate a transition to adulthood. Arnett also expresses how emerging adulthood is distinct for identity exploration, claiming this period of life offers the greatest opportunities for identity exploration, particularly in relation to work, love, and worldviews.

Despite the clear applicability of emerging adulthood to youth in our Canadian research context, Arnett stresses that emerging adulthood is not necessarily a cross-cultural phenomenon; rather, it is “a period that exists only in cultures that postpone the entry into adult roles and responsibilities until well past the late teens” (2000, p. 478). Socioeconomic status may also impact who develops through emerging adulthood, such that members of lower socioeconomic strata may lack opportunities for the explorations provided in emerging adulthood, needing rather to immediately transition from adolescence into adult roles (10).

#### 1.2.1. Mental health determinants and barriers to treatment in emerging adulthood

Barriers to adequate mental health treatment are alarmingly frequent in youth, and begin before the stage of emerging adulthood has even been reached. Early risk factors for developing significant mental health difficulties include individual factors such as neurochemical imbalances; adverse life experiences, such as abuse or poverty; family dysfunction, such as exposure to domestic violence; social factors, such as peer rejection; and community characteristics, such as a lack of access to recreational facilities and support services (7, 11). Poverty and low socioeconomic status have been found to be highly correlated with negative mental health outcomes (7, 11), particularly in cases where a lack of financial stability bars the path to post-secondary education, financial independence, and opportunities for identity exploration (11). The latter two barriers are of particular concern as Arnett (1) defines financial independence and opportunities for identity exploration as vital for healthy development in emerging adulthood.

Social disadvantages affecting the prevention and treatment of mental health issues are even more prevalent in certain young populations, including Indigenous populations, racial and ethnic minorities, LGBTQIA2S+ (lesbian, gay, bisexual, transgender, queer, intersex, asexual, two-spirited, and other non-cisgendered nor heterosexual) persons, youth who are street-, justice-, or child welfare system-involved, and persons with disabilities (7). MacLeod and Brownlie (7) identified a list of factors influencing mental health concerns for various populations transitioning into adulthood, including: reduced access to opportunities and resources; barriers to autonomy; harassment, discrimination, and oppression; trauma and maltreatment (particularly among women, who experience significantly more assault during emerging adulthood than men), street- and child welfare-involved youth, and Indigenous populations who are exposed to the impacts of intergenerational trauma and a lack of access to services. Harassment, discrimination, and oppression are particularly prevalent for LGBTQIA2S+ youth who often reach emerging adulthood after having experienced high levels of stress from hostile experiences in both educational and home environments (7). People of minority ethnic backgrounds and Indigenous populations are also heavily impacted by discrimination, as youth from these groups experience racism and discrimination, while Indigenous populations must also face an historical context of oppression and trauma in Canada (7). A lack of access to the proper mental healthcare services is particularly common in justice- and street-involved youth, as services for youth in these situations become less available as they progress through emerging adulthood into adulthood (7). Additionally, Indigenous populations and other cultural groups may not share the worldview of the healthcare treatment services available to them, possessing a less medicalized orientation to treatment (7).

Neighborhood environment also appears to be a significant determinant of mental health in emerging adulthood, with neighborhood attributes such as socioeconomic disadvantage, instability, lack of social cohesion, and contexts of considerable income inequality all linked to depression (12). These attributes arise from a lack of investment in–and limited resources for–positive health treatment and promotion in disadvantaged neighborhoods, and depression may further arise because of exposure to interpersonal violence and other stressful life events in disadvantaged neighborhoods lacking the proper supports (12).

Mental health literacy, defined by Gagnon et al. (6) as encompassing both knowledge and beliefs about mental health disorders, appears to be limited in emerging adults and poses yet another hurdle to wellness in these transitional years (4, 6, 8). Bluhm et al.’s (4) Canadian research found that participants were unsure if their mental health issues were “normal” feelings pertaining to external factors or if their symptoms were more serious, and many participants also expressed that they had negative mood and anxiety experiences for a long time before seeking help due to uncertainty about what was “normal” versus unhealthy. These data demonstrate a lack of knowledge in emerging adults in relation to what constitutes a “normal” mood range. Australian research into young people with mental health problems adds additional insight into the lack of mental health literacy in emerging adults: McCloughen et al. (8) found that young adults, while having a partial understanding of the mental health supports that general practitioners offer, believed that general practitioners’ supports and services lack the ability to significantly impact their health. One participant, for instance, believed check-ups by general practitioners did not have any significant impact on their health, and others expressed that they would only see a general practitioner if their *physical* symptoms were severe (8). This research also determined that low health literacy is associated with poor decision-making in patients, and that young people with mental illness possess limited information regarding healthcare treatments. In turn, low health literacy leads to treatment hesitancy, especially in relation to medications (particularly due to concerns about negative side effects).

Back in the Canadian context, Gagnon et al.’s (6) research into university students’ knowledge of mental health resources, attitudes toward seeking mental health treatment, and familiarity with mental health warning and action signs found that emerging adults had inadequate knowledge about mental health services. In particular, the students demonstrated a lack of knowledge about how to access services and associated service costs (6). Further data revealed that participants held insufficient knowledge of on and off campus mental health services, including not knowing about campuses’ free mental health services; many participants reported not knowing which services were available, not knowing where to go to access services, and not knowing how to schedule service appointments as being significant barriers to their mental healthcare (6).

Stigma surrounding mental health issues and treatment is also a major barrier to accessing mental health services (4, 6, 13, 14). Bluhm et al. (4) define stigma as the perception of others’ negative attitudes and the internalization of negative beliefs held by a society that affect a person’s attitudes toward themselves, p. 779. Feelings of shame and embarrassment–as well as fear of judgment from others–are by-products of stigma (6), and fear of stigma among emerging adults with mental health concerns extends to fears of being judged not only by peers, but by family and even health practitioners (4). Individuals who experience stigma from their peers tend to withdraw socially (4), and stigma associated with mental disorders may persist even when there is a biological basis to the disorder.

Other barriers to help-seeking for mental health concerns include: negative beliefs about help-seeking, preference for self-reliance, and poor recognition of one’s own mental health decline (6); a fear of a lack of control over one’s healthcare, such as an inability to choose a healthcare provider or forms of treatment (4, 6); increasing mental health symptoms, which decrease intentions to seek help (6); general practice-based services often being unable to meet the mental health needs of emerging adults (8); lack of social support (11); and having multiple risk factors, such as being a member of the LGBTQIA2S+ community and having low socioeconomic status (11). Certain dispositional traits have also been found to be barriers: for example, emerging adults are typically reactive rather than proactive with regard to mental health concerns, meaning that emerging adults will often wait until mental health warnings and action signs become extreme and difficult to hide from others before seeking help (6). Additionally, dispositional optimism, which is the general expectation of positive outcomes, is inversely related to mental health problems, while maintaining a negative cognitive style is often a precursor to increased mental health concerns and a reduction of health seeking behavior (11).

### 1.3. The Canadian healthcare system

#### 1.3.1. Organizational framework

The Canadian Constitution largely determines the organization of the Canadian healthcare system, dividing roles and responsibilities between the federal, provincial, and territorial governments (15). The federal government’s main responsibilities include setting and administering national healthcare policies under the Canada Health Act, offering financial support to the provinces and territories, and delivering primary and supplementary services to certain groups of people such as Indigenous populations living on reserves, Inuit populations, eligible veterans, and some groups of refugees (15). The Canada Health Act includes criteria and conditions for health insurance plans that must be met by all provinces and territories, requires provinces and territories to provide reasonable access to medically necessary hospital and doctors’ services, and discourages extra billing and user fees (15–17). If these criteria are not met, provinces and territories may not receive full federal transfers for health support. The federal government is also responsible for health protection and regulation including “the regulation of pharmaceuticals, food and medical devices” [(15), the federal government section, para. 5], consumer safety, disease prevention and surveillance, providing support for health promotion and research, and instituting health-related tax measures (15). Provincial and territorial governments plan and administer most of Canada’s healthcare services, with the caveat that these governments must follow the policies set out by the Canada Health Act (9, 15).

#### 1.3.2. Where might problems lie?

The federal, provincial, and territorial governments of Canada worked together in 2003 to create the Action Plan for Health System Renewal and the First Ministers’ Accords of 2004. With input from governments, care providers, and citizens, the policy work aimed to ensure annual and comprehensive public health reporting using pre-determined indicators of health status, outcomes, and service quality (18). Having these systems in place was thought to prove beneficial as “public performance reporting may increase accountability, enable public participation in healthcare (19, 20), impact societal and professional values surrounding our healthcare decision-making, direct attention to issues not currently on the policy agenda (21, 22) and improve [healthcare] performance” (18), p. 96. However, this 10-year mandate expired in 2014 (23). Following the expiration, Johnston and Hogel (18) thus chose to review the status of performance reporting in primary healthcare (i.e., healthcare services that constitute a patient’s first point of contact with the health care system). This included reporting on: access to care; the composition of groups providing care; patient satisfaction with care; and the degree to which technology is being used and incorporated into the primary care system. Johnston and Hogel (18) begin by explaining that Canada does not have a single primary healthcare system, but rather 13 distinct systems run by each individual province and territory, following the policies indicated in the Canada Health Act.

From the review, it was found that no province met the reporting requirements on the performance of their primary healthcare system; in fact, certain provinces, such as Saskatchewan, did not report at all between 2008 and 2012 (18). More than 5 years after the agreements were made in the Health Accords, no province or territory, nor the federal government, met all of their obligations. Johnston and Hogel (18) further note the distressing fact that, even up to the time of their study, provinces continued to use different data sources and primary healthcare indicators, such that it prevented interprovincial comparison of indicators. Their research demonstrates a lack of cohesion between federal, provincial, and territorial governments in maintaining and applying healthcare policies in Canada, and of particular concern, it demonstrates a lack of foresight in maintaining proper healthcare reporting.

The barriers to proper mental health treatment in Canada that arise from the lack of cohesion and planning denoted by Johnston and Hogel (18) are highlighted in the work of Moroz et al. (13), who demonstrate the significant need for improved mental health services in Canada. In 2018, 5.3 million Canadians reported needing mental health services within the previous year; however, only 21% of people reported that their needs were fully met when they sought out such services (13). Previously documented service barriers in Canada included lack of accessibility of services, lack of knowledge about where to go, language barriers, geographic and demographic inequities (e.g., rural communities and Indigenous populations who are often far removed from large population centers where most mental health resources are located), and a lack of government oversight (13). They also found that 78.2% of the most frequently reported barriers had to do with personal circumstances such as being too busy, not knowing where to go, or being unable to pay for services. For example, clients deemed ‘eligible’ can receive up to 22 h of counseling services every 12 months covered by the Government of Canada. However, beyond those 22 h all coverage is provided on a case-by-case basis (24) and Moroz et al. (13) found that 30% of Canadians have to pay out of pocket for private practice psychotherapists. Stigma was also a highly reported barrier to accessing mental health services in Canada, with many Canadians reporting feeling devalued by health professionals (13). Additional issues in accessing mental healthcare that they found include a lack of access to service in rural communities; long wait times for counseling, particularly when it is needed for children and youth; and a heavy reliance (80%) on family physicians to meet Canadians’ mental health needs with only 23% of these physicians feeling prepared to serve mental health problems. Financially, mental illness and mental health problems cost Canada $51 billion annually in 2003 (25) which equates to over $75 billion in 2022 (26), and care for mental health conditions is estimated to be underfunded by $3.1 billion annually. Moroz et al. (13) conclude that while there are evidence-based programs that are designed to improve mental healthcare outcomes and services, such as community-based service delivery, e-mental health initiatives, and psychotherapy, many of these methods have yet to be properly or fully integrated into the Canadian healthcare system.

Based on this overview of the context, we expect that a lack of mental health literacy, stigma, and lower average socioeconomic status will be seen to pose significant barriers to seeking mental health treatment in emerging adults. Additionally, we anticipate that the lack of cohesiveness between the federal, provincial and territorial governments of Canada will pose a major barrier in providing proper mental health resources and treatment to emerging adults.

### 1.4. Comparisons with other Western countries

When considering this research on a broader scale, it is relevant to note that other westernized countries have similar issues pertaining to emerging adult healthcare access and use as Canada. For example, Australia’s mental healthcare system has been deemed complex, with supports received varying widely depending on the pathway in which a person enters the system (27). In addition, co-payments and other cost sharing policies may deter low socioeconomic households from accessing mental healthcare (28). There are also limited mental healthcare specialists in rural and remote areas of Australia, such that may create difficulties for rural residents to access services they need (27, 29). Additionally, Saxby et al. (30) determined that groups who experience prejudice and discrimination in Australia, such as sexual minorities, may have additional difficulties accessing mental healthcare.

Research performed both in Australia and the United Kingdom has determined social and cultural factors acting as barriers to the use of mental healthcare. Some of these barriers include: stigma, negative perceptions surrounding mental illness and mental healthcare, and the desire for self-reliance (31, 32). Triliva et al.’s (33) investigation into healthcare professionals’ perspectives on mental health services in six European countries with varied welfare state provisions and health care systems (Belgium, Sweden, Norway, the Netherlands, Greece, and Cyprus) found that gaps in mental healthcare were comprised of numerous barriers. The main barriers include: funding, accessibility and availability of services, and human resources capacity. Regarding healthcare systems in Europe, transitioning from child to adult healthcare can be complicated, with only Denmark and the United Kingdom having guidelines for the transition process (34).

## 2. Methods

With the national policy context and broad social-psychological research contexts in mind, we then performed a focused, systematic literature review of primarily peer-reviewed qualitative and quantitative literature pertaining to young adult mental healthcare in Canada. We also conducted a gray-literature search of Government of Canada policies and documents regarding the mental healthcare system and emerging adulthood. Our main resource for finding literature was the online library catalog available to students and faculty at our institution, with results being further narrowed through specific databases including PubMed, APA PsychInfo, and Government of Canada websites. Our literature search focused on articles spanning from 2000 until present that pertain to the mental health of emerging adults; we focused on their experiences within the Canadian healthcare system along with the system’s policies regarding mental health treatment. The key information analyzed related to the Canadian healthcare system’s policies set in place at the federal level, as well as information about the administrative format of healthcare delivery (e.g., the structure of provincial and federal healthcare systems and relevant bureaucracies, to identify how the responsibility for specific policy-making and resource allocation is distributed).

The keywords used in this first search were: Canada, healthcare, mental health, government, policy, and administration. They were chosen as we believed they would best narrow our search to the most relevant resources pertaining to the Canadian healthcare system’s mental healthcare setup. The key information searched for in relation to emerging adults’ experiences was *emerging adults’ knowledge and understanding of mental health issues*, measured by an emerging adult’s general, logistical (e.g., organization of health resources and policies), and procedural (e.g., how to access and utilize mental health resources, what to expect) mental health knowledge, as well as their ability to recognize signs of mental health disorders, and factors that influence emerging adults to either access or reject mental health services. The keywords used in this second search were: emerging adults, young adults, mental health, healthcare, knowledge, experience, and Canada. These keywords were chosen to narrow our search to the topic of interest and to limit our search to Canadian experiences whenever possible. Because the catalog we utilized contains hundreds of thousands of documents, we also included strict inclusion criteria to narrow our search.

### 2.1. Inclusion criteria

Documents included in the in-depth analysis had to meet one of two sets of parameters. For the first set, comprising academic publications, articles had to be theoretically and/or empirically focused on the emerging adulthood period, and span from 2000 to the present. This publication range was chosen to parallel the period when the concept of emerging adulthood (1) became prominent in the relevant literature. For the second set, comprising gray material (i.e., non-peer reviewed technical reports) criteria included a focus on emerging adults’ mental health experiences, namely having, understanding, and/or treating mental health disorders, as well as emerging adult knowledge and experiences related to seeking mental health treatment. These articles had to have been published from 2007 until present. We set a span of 15 years for this set to allow us to analyze sufficient data while ensuring that the reports were up to date with current Canadian healthcare policies. In this second set, articles relating to Canadian healthcare had to include information on federal and provincial healthcare policies, mental health resource distribution, and/or action plans for furthering mental healthcare and treatment. We only included reports whose policies were the most recent, as compared with policies on Government of Canada websites. Initially, 3,611 were returned through our search methods, and were further reduced through the specification of our inclusion criteria and through the exclusion of articles that focused on specific medical conditions (e.g., the experiences of cancer patients) or healthcare systems outside of Canada (see Appendix A). The resulting corpus of documents included 18 academic publications and 10 technical reports, for a total of 28 documents, summarized in Appendix B.

### 2.2. Data analysis

We performed a thematic analysis using Braun and Clarke’s (35) six phases to determine themes pertaining to issues in Canadian healthcare vis-à-vis emerging adulthood. The six phases of Braun and Clarke’s (35, 36) thematic analysis approach that we followed are: (1) *Familiarization with the data*, performed throughout our systematic review. In this phase, we took notes for coding, marking ideas commonly seen throughout the literature that could be good candidates for themes. (2) *Generation of initial codes*, which continued the data analysis as we systematically worked through generated ideas and relevant data and developed them into codes that identified a semantic (directly relating to a topic or meaning) or latent (relating to a topic or meaning not blatantly manifested) feature of the data. We then organized our data into a few meaningful groups that would be used to develop broader themes. (3) *Search for themes*: From the coded data, we next generated themes. Codes were sorted and combined into overarching themes and potential subthemes. By the end of this stage, we had a collection of potential themes and subthemes that were organized into a thematic map. (4) *Review of themes*: This phase included refinement of potential themes and subthemes from the previous stage. Some themes were collapsed into each other to form a single theme, while other themes were broken down into separate themes. This phase included analyzing themes to see if they formed a pattern or if any themes were outliers (we must consider the validity of individual themes in relation to our data to ensure themes accurately reflect collected data). The final themes and subthemes were organized into an updated thematic map. (5) *Definition and naming of themes*: Once a satisfactory thematic map was produced, we identified the crux of themes–individually and as a whole–and determined which aspect of the data each theme captured. We conducted and wrote a detailed analysis of each theme, considering how each one fits in relation to the other themes and into our overall data. This phase of the thematic analysis also acted to solidify which themes contain subthemes, and what those subthemes were. We concluded this phase by giving concise names to our themes so readers can immediately identify the essence of each theme and subtheme. Finally, (6) *Production of the report*: Once we had a full set of critically analyzed themes, we began writing our final report.

Regarding roles in the coding and data analysis process, the first author developed the initial coding frame of codes, overarching themes, and subthemes identified the content of all articles. The second author then reviewed, both independently then in discussion with the first author, the coding frame and suggested refinements in the scope and precision of the frame’s dimensions. The first author then adjusted the coding frame until both researchers came to a consensus that the coding frame was satisfactory in its scope and its clarity.

## 3. Results

### 3.1. Barriers to mental healthcare

#### 3.1.1. Personal circumstances

Numerous personal circumstances appeared repeatedly throughout the literature as barriers to emerging adults’ mental health seeking (4 articles). These barriers, in order from most to least common, include: fears of mental health costs being unaffordable [c.f., (13)]; negative beliefs about help-seeking [c.f., (6)]; a preference for self-reliance [c.f., (4)]; and being too busy [c.f., (13)]. Many emerging adults, due to these barriers, will avoid primary healthcare services and hold beliefs that general practitioners do not offer proper supports for mental illness.

#### 3.1.2. Stigma

Stigma was a heavily reported barrier to accessing mental healthcare among emerging adults, appearing both directly and indirectly in over half of the literature pertaining directly to emerging adult health needs (with 8 articles speaking directly to stigma). There was a persistent trend throughout the literature of emerging adults feeling uncomfortable, and sometimes even unsafe, speaking about their mental health problems to health care providers. Emerging adults also noted feelings of shame and embarrassment regarding accessing mental healthcare and expressed a fear of judgment–not only from family or peers–but also from healthcare providers. Additionally, emerging adults were concerned about a lack of privacy and confidentiality in accessing mental healthcare. For example, Bluhm et al. (4) had several participants in their study express fears of being seen differently if it was known they were accessing mental healthcare, with one 19-year-old female participant saying “[h]igh school and teenagers is all about like putting you into like a pinhole [*sic*] and it’s like, that’s who you are and I do not want to be like ‘the crazy girl’ at [name of school]” (p. 783).

#### 3.1.3. Mental health literacy

Poor mental health literacy was a common theme pertaining to barriers to mental healthcare, appearing to some degree in well over half of all literature pertaining to emerging adults (with 3 articles directly using the term “mental health literacy”). Young adults’ poor recognition of mental health decline and of which mental health symptoms indicate a need for treatment was particularly detrimental to help-seeking, as emerging adults lacking this knowledge will seldom seek mental health treatment unless their symptoms become severe (e.g., suicidal ideation, violence toward others, impairment that cannot be managed on one’s own) or difficult to hide. A lack of knowledge of mental health services or how to access these services was also commonly noted as being a barrier to help-seeking. Specifically, knowledge pertaining to costs, locations, eligibility requirements, and treatment expectations seems to be lacking in emerging adults. For example, in Gagnon et al.’s (6) research, the authors found that while “university counseling [*sic*] services are free to students, results revealed that not all students were aware of the free mental health services available on campus as they identified constraints on finances as a critical barrier to accessing mental health services” (p. 655).

#### 3.1.4. Accessibility

A lack of accessible services was emphasized as being a barrier to mental healthcare seeking, appearing in 9 articles. Accessibility restrictions included long wait times, which can be detrimental for emerging adults; in short, “wait times for counseling and therapy are generally too long for effective care when it is needed most…. In [the province of] Ontario, wait times of 6 months to 1 year are common” (13), p. 283. Additional restrictions include concerns over service costs which make accessing mental healthcare difficult for emerging adults of lower socioeconomic status. This concern over costs may be exasperated among University students, with tuition costs at Canadian Universities having doubled between 1990 and 2005 (37). Emerging adults living in rural areas also face difficulties in accessing mental health supports which are often localized to larger population centers.

#### 3.1.5. Social support

A lack of social support was found throughout the literature to be a major barrier to mental healthcare seeking, with the importance of social support appearing in 11 articles. Parental, peer, and romantic connections are all important supports in emerging adulthood. However, a lack of these supports from these connections, particularly if these connections demonstrate negative views toward mental health treatment, will deter emerging adults from accessing mental healthcare. Many emerging adults are also concerned that receiving mental healthcare may put a strain on their relationships, such that doing so may lead to social disadvantages like peer rejection [c.f., (4)].

### 3.2. Mental healthcare concerns in Canadian healthcare policy and services

#### 3.2.1. A lack of cohesion

Health expenditures in Canada vary heavily across provinces and territories, with the services each region offers being dependent on demographic factors, geography and population density, and population age (as emphasized in 7 articles). Additionally, coverage levels and mental health action plans vary across the country, with each province and territory having their own goals regarding mental healthcare. There is also a noticeable lack of collaboration between healthcare services, including mental health services, addiction services, and primary care providers, such that it restricts the delivery services and supports required by emerging adults (18, 38).

#### 3.2.2. Issues in the primary healthcare system

Issues within the primary healthcare system appear in 7 articles. The primary healthcare system has been reported to be lagging behind all other health system sectors (18) and harbors numerous problems that act as barriers to accessing mental health services. These barriers include: a lack of accessible information regarding mental healthcare; long wait times to access mental health resources; a shortage of accessible mental health professionals; a lack of government oversight; stigma; geographic inequity (e.g., rural areas often do not have mental health resources or facilities readily available and require those seeking treatment to travel to larger population centers); and demographic inequity (e.g., many mental health services are not designed with cultural sensitivity in mind, often leading to diverse populations’ lack of access to healthcare facilities and facilitators that understand their unique sociocultural needs). The primary healthcare system is not set up well to assist diverse populations, and many emerging adults of diverse backgrounds may struggle to find the proper care of physicians to meet their needs. As acknowledged by the Mental Health Commission of Canada (38), “many youth and emerging adults can face difficulty finding service providers and professionals that share their racial, ethnic, religious, cultural and linguistic backgrounds and culturally safe and appropriate support can be hard to find.” It is important to note that many primary healthcare physicians do not feel adequately prepared to handle mental health concerns, and few supports or resources are offered to these physicians. Supports are also lacking for patients who do not qualify for supplementary benefits under government plans, and many emerging adults have found themselves paying out-of-pocket for mental health services as many mental health services are not covered. Typical Canadian coverage “ranges from only $400 to $1,500 annually, covering only 2–8 therapy sessions” (13), p. 282.

#### 3.2.3. Emerging-adult-specific concerns

Emerging-adult specific concerns appeared within 5 articles. Many emerging adults who were engaged in child and youth mental health services (including but not limited to child welfare and the youth justice system) “age out” of the provided care system without adequate supports. In fact, the literature pinpoints a lack of government and associated program supports that assist in the transition to the adult healthcare system: “Unfortunately, because [emerging adults] are often forced to transition out of youth services around the age of majority (18–19, depending on the province or territory), many emerging adults are not able to access vital services and programs at a time when they need them most” (39). There is also a distinct lack of a nation-wide approach to transition management or clinical service delivery approaches for emerging adults, and no national strategy or policy guidelines for emerging adult mental health exist. Emerging adults are not yet seen as a distinct population for policy, planning, funding, or service delivery, and no province or territory has fully implemented any emerging adulthood protocols (40).

#### 3.2.4. Indigenous concerns

Indigenous considerations appeared in 4 articles. Past and ongoing colonialism has disrupted the mental health of many Indigenous peoples. Indigenous populations are reported to face inequitable circumstances that can impact mental health and transitions across healthcare systems, particularly into adulthood. Many Indigenous emerging adults also do not have access to culturally safe mental health programs and services that respect the beliefs and traditions of Indigenous populations. Details pertaining to exactly what would constitute culturally safe programs were vague; however, the Mental Health Commission of Canada did offer some insight into what culturally safe practices might look like in relation to Métis peoples:

Like other Indigenous peoples, Métis people view wellness, including mental wellness, holistically, and possess inherent knowledge of how to heal themselves. It is therefore important to approach wellness from a Métis-specific lens and recognize that there are distinct, traditional Métis ways of knowing that Métis people can draw from to get and stay well (38).

In addition, many Indigenous emerging adults live in remote and rural regions, such that geography limits accessibility to most mental health services and supports (38).

#### 3.2.5. Rural concerns

Rural-specific concerns appeared in 3 articles. Emerging adults living in rural areas face additional barriers to accessing mental healthcare. Particularly, a lack of both primary care and specialized care means emerging adults in rural areas are cut off from many of the services and educational opportunities necessary to better their mental health (13, 40). Emerging adults who require intensive mental healthcare must often be transported out of these rural areas and away from their communities, increasing the risk that they will avoid accessing mental health supports when necessary.

### 3.3. Removing barriers to mental healthcare

#### 3.3.1. Increased access to mental health services

Increasing access to mental health services was highly indicated as the best way to boost emerging adult use of mental health services. Suggestions to increase access to mental health services in Canada were found in over 50% of all articles reviewed (expressed explicitly in 7 articles), excluding Arnett’s works (that do not focus on the Canadian context) and government documents describing federal and provincial policies. Increasing service delivery efficiency was recommended, particularly by utilizing online, or e-health, services. Using technology and online resources is natural for emerging adults, given that they have grown up in a context of widespread e-resources, and e-mental health interventions have shown promising results in increasing mental health service access (13, 14). E-mental health services also increase confidentiality, which has been highlighted as important to emerging adults (4, 40). Additionally, the COVID-19 pandemic has increased the production of virtual care solutions, thus encouraging an overall shift toward more e-health options.

The use of other evidence-based mental health delivery options may also increase accessibility to mental health services. Community-based delivery services have been shown to be effective in providing mental healthcare access and delivering early interventions while reducing wait times. Expanding psychotherapy services may also prove effective at reducing medical costs, both for patients and for the Canadian economy; in fact, “Evidence shows that, in Canada, every dollar invested in psychotherapy would save two dollars to society” (13). Additionally, increasing the flexibility of mental healthcare would be beneficial by expanding collaboration among sectors (educational, medical, social, religious, and community setting) to offer easier accessibility to mental health education and resources.

#### 3.3.2. Facilitators to formal help-seeking

Social support (found in 11 articles) was noted as a major facilitator to formal help-seeking among emerging adults; specifically, insistence from friends or family that one seek care, as well as having good relations with healthcare providers (4, 6). For example, in Bluhm et al.’s (4) research, “participants commented that urging and insisting from their family or friends that they seek help would facilitate access to services” (p. 655). Additionally, social participation may increase positive mental health outcomes (41). Improving emerging adult knowledge of mental health services (e.g., how to access services, the process of therapy) and knowledge of mental health symptoms were also highly recommended to facilitate mental healthcare participation in emerging adults.

Guaranteed confidentiality (discussed in 5 articles) or privacy when accessing mental healthcare was indicated as being an incentive to accessing mental health services. In general, emerging adults prefer decreased visibility from the public eye, and suggested increasing the ability for those seeking care to book appointments at allied health centers rather than solely at specialized clinics (6). Additionally, emerging adults expressed a desire to be active in their own healthcare, exerting control as a facilitator of their own mental health treatment. Two paths in which greater control can be offered to emerging adults are: (1) increasing mental health literacy in emerging adults, and (2) having healthcare providers work with emerging adults, expanding their understanding of their own mental health and developing plans of action to treat negative mental health symptoms, rather than simply providing medication.

Finally, reducing stigma or normalizing mental healthcare were frequently raised as being factors that encourage mental healthcare participation in emerging adults (found in 7 articles). Both can be achieved through early mental health education and through the promotion of logistical and procedural information regarding formal help-seeking. In particular, stigma can be reduced by normalizing mental health as a general component of overall health: “Professionals and educators should consider promoting awareness about the implications of failing to seek help in response to warning signs. Moreover, instruction on accurately appraising mental health symptoms should be incorporated into the education system curriculum” (6).

### 3.4. Improving the Canadian mental healthcare system

#### 3.4.1. Federal funding allocation

Numerous federal funding recommendations were made throughout the literature reviewed. Suggestions include increased funding to sustainable, evidence-based mental health services such as e-health and psychotherapy, which was further suggested to be covered under universal Medicare in Canada. As an example, the Canadian Alliance on Mental Illness and Mental Health (CAMIMH) suggested the “federal government increase its cash contribution to the provinces and territories by a minimum of $277.5 million a year to improve timely access to mental health services” (42), p. 6. Another example includes a suggestion to increase the amount of health spending devoted to mental health from 7 to 9% over a period of 10 years (43). It was also suggested that funding be allocated to various mental health institutions, such as the Canadian Institute of Health Information (CIHI) and the Canadian Institute of Health Research (CIHR), and to establish a centralized, national mental health body. Finally, it was recommended that funding be heavily allocated to the education system, beginning at the earliest stages, to help foster mental health literacy and resilience in emerging adults.

#### 3.4.2. Federal policy changes

Some suggestions were made throughout the literature with regard to federal changes at the policy level. Primarily, suggestions were made for the federal government to create a national standard for accessing mental health services, either by amending the Canada Health Act or by the introduction of a new federal Mental Health Act as no act of this type currently exists at the federal level (42). A second suggestion was to establish policy ensuring Canada-wide competencies for professions working in the field of emerging adulthood mental health, both to ensure proper formal training but also to ensure consistency in treatment methods nation-wide. Finally, it was suggested to re-examine policy that separates adolescence and adulthood at the age of 18 (44), and to recognize emerging adulthood – and its age range – as a unique developmental period in life. These last two suggestions were first expressed by a panel of emerging adults and a jury of mental health policy makers at the consensus conference for emerging adults (38). By recognizing this demographic, policies, programs, and services can be created and delivered on the basis of need rather than age (which typically is separated to child, youth, and adult categorizations), and nationally funded, evidence-based practical and clinical guidelines pertaining to emerging adult mental health can be formed.

#### 3.4.3. Changing the mental healthcare system

Suggestions were made to change the current Canadian mental healthcare system. It was recommended that all provinces and territories should work collaboratively with the federal government to develop up-to-date public and private health expenditures in mental health reporting, as well as establish a national emerging adulthood mental health initiative in joint partnership with emerging adults, who are experts in their own care:

Collaborate in joint partnership with emerging adults as experts in their own health care to define outcomes and determine the most appropriate services to meet their needs. Remove barriers to collaboration and integration of services and sectors to ensure continuity of mental health care for all emerging adults. Dedicate time and funds to improve evaluation, data collection, research and knowledge exchange in emerging adult mental health (45).

Notable changes to the mental healthcare system also included ensuring equity among diverse populations (38, 40). Principles of health equity, anti-oppression, intersectionality (i.e., recognizing the compounded effects of social locations), and socio-cultural contexts must be taken into account when creating policies and developing mental health structures for emerging adults. The Mental Health Commission of Canada (38) argues for these principles, reasoning that “marginalized [emerging adults] with mental health problems get stuck in an endless cycle of being marginalized” (p. 14), with culturally safe and competent support (e.g., providers that share cultural backgrounds with patients) often being difficult to locate.

## 4. Discussion

Our in-depth review aimed to identify the major barriers emerging adults faced in seeking mental health treatment, with a particular focus on the Canadian healthcare system. From this research, trends were identified pertaining to the most common barriers to seeking mental health treatment for emerging adults, where the Canadian healthcare system may be lacking in this regard, and steps that may be taken in order to rectify these barriers.

In terms of mental health barriers, our systematic review revealed some critical findings. Three of these barriers include low mental health literacy, stigma, and costs associated with services not covered under basic Medicare, confirming our hypotheses on this front. A primary barrier to accessing mental healthcare among emerging adults was a lack of mental health literacy. In line with prior research (4, 6, 8), a lack of knowledge pertaining to mental health resources, mental health symptoms, and mental health treatment options proved detrimental to the mental wellbeing of emerging adults.

A particularly distressing finding was that many emerging adults would only seek mental health treatment when faced with the most severe or debilitating symptoms (including, but not limited, to: aggressive behavior, suicidal ideation, and self-harm). This underscores an immediate and focused need for increased mental health education and promotion of mental health resources directed at emerging adults, and it further suggests that there may be a far greater number of emerging adults facing problematic mental health symptoms than are being reported at mental health clinics and care centers.

Stigma was another heavily reported barrier to accessing mental healthcare in our analysis—a finding that bolsters prior findings [e.g., (4, 6)]. Feelings of shame and embarrassment were salient factors contributing to the stigma surrounding help-seeking, in addition to concerns that emerging adults may be judged negatively by family, peers, and healthcare professionals. While socioeconomic status, discussed by MacLeod and Brownlie (7) in our broad literature review, was only somewhat relevant in our focused document analysis, concerns over mental healthcare costs were repeatedly found throughout our focused analysis to be a major barrier to emerging adults accessing mental health resources.

Social factors were not heavily discussed within our initial literature review, but were quite relevant in our subsequent systematic review. Support from family and peers was found to be a powerful contributor for emerging adults to seek mental healthcare if these connections had positive views on help-seeking, but so-called support turned out to be a barrier if these connections had negative views on help-seeking. In addition, support from–and positive relationships with–healthcare providers were also deemed important by emerging adults, though many emerging adults raised concerns about not being able to develop a good rapport with mental health practitioners, or the practitioners not offering the proper support a specific individual needed. This underscores a need for greater accessibility to a variety of mental health service providers to ensure emerging adults have as many options as they need to find the proper treatment and treatment facilitator for their unique needs.

While the initial literature review showed evidence of negative mental health outcomes for diverse populations (e.g., LGBTQIA2S+ individuals and persons of color), not much information pertaining to these populations in the Canadian context was determined from our systematic review. However, we did not explicitly search for these factors, and as such, there may be deeper trends pertaining to diverse emerging adult populations and accessing mental healthcare yet to be discovered.

Regarding problems in the Canadian healthcare system, a distinct lack of cohesion plays a major role in complicating or outright barring the path to mental health treatment among emerging adults. This lack of cohesion within Canadian government policies, bureaucracies, infrastructure, and services validates our hypothesis that the lack of cohesiveness between the federal, provincial and territorial governments of Canada will continue to pose a major barrier in providing proper mental health resources and treatment to emerging adults. Of particular note, the lack of a strict federal guideline for what mental health services and policies must be provided has led to an interprovincially and inter-territorial disconnect on which services are offered in each region, and how mental healthcare is covered regionally. This reflects a pronounced lack of communication and coordination between the federal, provincial, and territorial governments relating to mental healthcare nationwide, and reflects the findings of Johnston and Hogel (18) who found that no province, territory, or federal government met all of the reporting obligations set for them from the Health Accords.

It should be noted that finding information pertaining to emerging adulthood, or at least the relevant age range (~18-26), among Canadian federal government sources was incredibly difficult, and required digging through numerous documents and reports, many of which included links that redirected to third party or Government of Canada affiliate organizations. This makes it clear that federal resources are not being adequately applied to this area of healthcare, and highlights a lack of organization and research, as well as a lack of funding, within both the federal and provincial governments. Policies and data should be made clear and readily available for any Canadian citizen seeking information regarding mental healthcare in Canada. However, to the credit of the federal government, locating mental healthcare facility resources (including addresses and contact information for various mental healthcare facilities) on federal websites was straightforward.

In terms of improving mental healthcare among emerging adults, giving emerging adults a sense of control over their own healthcare was emphasized as being important, and reflects prior research (notably (4, 6)). Results from our systematic review underscored a desire among emerging adults to have control over their own healthcare. Control may be offered, most importantly, to emerging adults by increasing their knowledge of mental health symptoms and resources. In fact, increasing mental health education and early intervention were repeatedly highlighted as the first steps that should be taken in order to improve emerging adult healthcare participation.

### 4.1. Future directions in emerging adult mental healthcare

Based on the results from this systematic analysis, we will now offer some suggestions for the future of the Canadian healthcare system with regard to caring for emerging adults’ mental health needs. We expect that these suggestions will be both applicable and appealing–to varying degrees–to other countries struggling with mental health management in emerging adults. First, changes to funding and policies to benefit emerging adults’ mental healthcare needs should consider the perspectives and needs of the emerging adulthood by first acknowledging this age range as a distinct developmental period, much like how Arnett (1) has. Funding should be directed to increasing mental health education, and policy should be put into place to mandate mental health education as early as primary school. Mental healthcare should also be covered by Medicare in Canada, and no Canadian should have to pay out of pocket or through private insurance for mental healthcare. Policies should also be put into place to ensure a Canada-wide set of standards pertaining to mental health procedures, clinical guidelines, and facilities offered to create consistency throughout the country. It is important to note, however, that mental health is in many ways a cultural construct, and as such we cannot expect a uniform national model to work for all populations–whether in Canada or elsewhere. It may therefore be beneficial, in the Canadian case and in other national contexts, to develop a federal board to work directly with diverse populations. Such a board could allow the facilitation of emerging adults voicing their concerns regarding their own healthcare, which has previously proven difficult (46). In addition, mental health system reporting measures, similar to those developed in the First Minster’s accords of 2004 (18) but which expired in 2014 (23), should be re-implemented nation-wide, with an obligation that they be fulfilled or met by provinces and territories in order to receive full federal funding. It may also be beneficial to offer opportunities for primary care physicians, and physicians in training, to increase their own mental health literacy to better aid and direct emerging adults seeking mental healthcare to appropriate resources. Beyond offering greater education opportunities for physicians and medical students, it may be advantageous to make mental health check-ups and screenings a part of all patients’ general yearly check-ups in order to normalize mental healthcare. Such check-ups could even be done remotely using e-health sources, which would not be too cumbersome to implement, and could reduce expenses for both patients and the healthcare system at large. The government of Saskatchewan has already taken action regarding this route of accessible healthcare, increasing funding for internet-delivered cognitive behavioural therapy programs as of a 2019 report (47).

Beyond the specific suggestions we put forth above, on a macro level our research offers greater insight into the barriers that emerging adults face in receiving proper mental health treatment in Canada. There are many potential ideas and implications for how our findings may be further applied. The findings from our systematic review could be utilized to better the mental health and treatment of emerging adults by demonstrating where young adults’ greatest causes of concern lie. Additionally, findings could be used to improve upon Canada’s current healthcare model at both the federal and provincial levels, such that they may offer suggestions for achieving a more cohesive and universal approach to combating mental health issues in emerging adults, rather than the current patchwork model with its federal, provincial, and interprovincial differences. Beyond using our findings to adapt current policies in Canada’s health model, they could be applied to Canada’s educational system as well through offering mandated courses on mental health issues and forms of treatment. We anticipate that this would improve the mental health literacy of young people such that it would normalize mental health concerns and treatments, and thereby better facilitate seeking help when it is needed–particularly during the tumultuous emerging adulthood stage. Most importantly, our analysis may act as a starting point or pilot test for future research, such that subsequent work may expand on the corpus of documents we collected and analyzed.

Our assessment does not come without limitations. In our systematic review, we were not able to fully compare interprovincial policies and administration. This, in turn, limited our ability to see the overall picture of possible province-to-province discrepancies in healthcare resources and delivery. Collecting and looking at data interprovincially–in extreme, granular detail–may offer an interesting opportunity to analyze the perspectives of emerging adults who have moved interprovincially and experienced multiple forms of mental healthcare. Of course, new sources and policies are always emerging or developing, rendering our findings representative of a moment in Canadian healthcare, but not necessarily or fully representative of a longer era’s situation and health care foci.

Future research could benefit from observing the current and historical context of the mental healthcare of each province and territory, as well as by analyzing the historical context of interprovincial and interterritorial healthcare relations. To expand this research even further, it may be fruitful for future research to compare Canada’s mental healthcare policies and administrations with those of other countries with universal healthcare.

## Data availability statement

The original contributions presented in the study are included in the article/Supplementary material, further inquiries can be directed to the corresponding author.

## Author contributions

JM conceived of the research idea in consultation with SK. JM created the sample, conducted the data analysis and wrote the first draft of the manuscript. SK revised the manuscript and added new sections to it and secured funding for publication of the manuscript. JM and SK worked together to develop an analytic strategy and collaborated to develop an organizational framework for the manuscript and contributed to manuscript revision, read, and approved the submitted version.

## Funding

Funding has been secured through an internal Article Preparation Grant from St. Thomas More College, University of Saskatchewan.

## Conflict of interest

The authors declare that the research was conducted in the absence of any commercial or financial relationships that could be construed as a potential conflict of interest.

## Publisher’s note

All claims expressed in this article are solely those of the authors and do not necessarily represent those of their affiliated organizations, or those of the publisher, the editors and the reviewers. Any product that may be evaluated in this article, or claim that may be made by its manufacturer, is not guaranteed or endorsed by the publisher.
