# Supplementary material for: Mental health treatment and access for emerging adults in Canada: a systematic review
Source: Front Public Health. 2023 Jul 10;11:1088999. doi: 10.3389/fpubh.2023.1088999 (PMC10370273; doi:10.3389/fpubh.2023.1088999)
Supplement: Supplementary file 1 [file Data_Sheet_1.docx]

**Appendix A: PRISMA Flowchart**


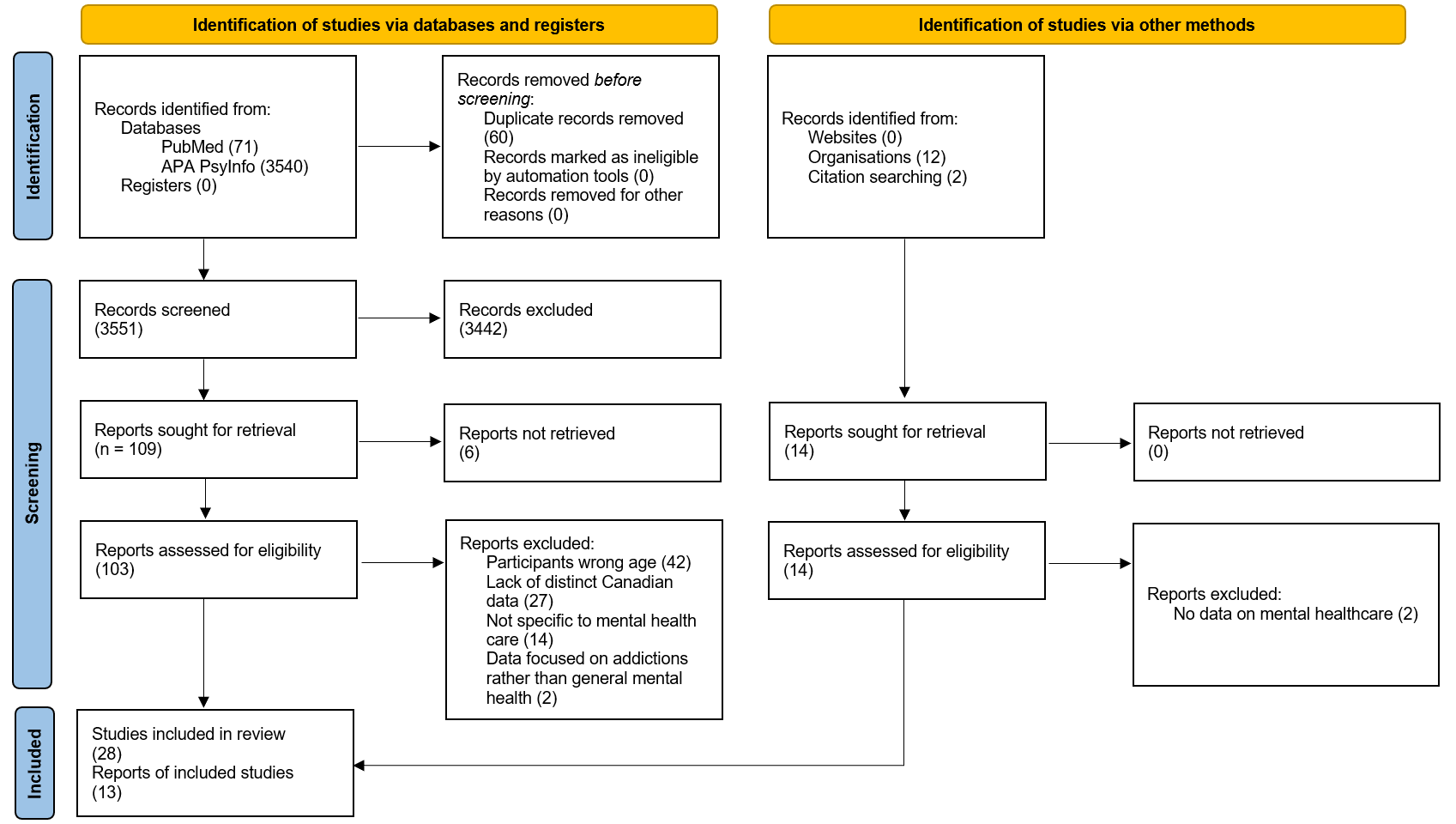


**Appendix B: Articles Table**

| Author Name(s) | Title | Year | Major Theme(s) |
| --- | --- | --- | --- |
| Bartram, M., & Chodos, H. | Changing directions, changing lives: The mental health strategy for Canada | 2013 | - Barriers to mental healthcare (stigma, cost); - Healthcare underfunding; - Recommendations for improving mental healthcare (education, services, policies, diverse supports) |
| Bluhm, R. L., Covin, R., Chow, M., Wrath, A., & Osuch, E. A. | “I just have to stick with it and it’ll work”: Experiences of adolescents and young adults with mental health concerns | 2014 | - Barriers (Stigma, mental health education, judgement by healthcare providers); - Recommendations for improving mental healthcare (control personal healthcare, improved mental health education, social support, confidentiality) |
| Canadian Mental Health Association | Mental health action plan: Better access and system performance for mental health services in Canada | 2020 | - Recommendations for improving mental healthcare (funding, policy change, further research) |
| Carver, J., Capelli, M., & Davidson, S. | Taking the next step forward: Building a responsive mental health and addictions system for emerging adults | 2015 | - Barriers (transitioning to adult mental health services, ageing out of services, accessibility); - Diverse populations concerns; - Federal cohesion or lack thereof; - Policy concerns; - Provincial/Territorial healthcare differences; - Rural concerns; - Recommendations for improving mental healthcare (inclusion of emerging adults in policy creation, improved reporting measures, policies) |
| Ferro, M. A., Gorter, J. W., & Boyle, M. H. | Trajectories of depressive symptoms in Canadian emerging adults | 2015 | - Impacts of social relations; - Impacts of socioeconomic status |
| Gagnon, M. M., Gelinas, B. L., & Friesen, L. N. | Mental health literacy in emerging adults in a university setting: Distinctions between symptom awareness and appraisal | 2015 | - Barriers (stigma, negative mental healthcare beliefs, self-reliance, low mental health literacy and knowledge of resources; fear of judgement, time constraints); - Help-seeking facilitators (Improved mental health literacy, positive social support, confidentiality, technological/online healthcare mechanics); - Reducing barriers (early mental health education, improved awareness of resources) |
| Gaudet, S. | Emerging adulthood a new stage in the life course: Implications for policy development | 2007 | - Academic impact; - Socioeconomic barriers; - Suggestions for emerging adult policy |
| Goldstein, R. B., Lee, A. K., Haynie, D. L., Luk, J. W., Fairman, B. J., Liu, D., Jeffers, J. S., Simons-Morton, B. G., & Gilman, S. E. | Neighbourhood disadvantage and depressive symptoms among adolescents followed into emerging adulthood | 2019 | - Barriers to mental healthcare (socioeconomic status, social relations, neighbourhood environment) |
| Government of Canada | Canada’s health care system | 2019 | - Barriers to mental healthcare (cost); - Demographic considerations; - Mental healthcare service Set-up/delivery; - Provincial/Territorial healthcare differences |
| Government of Canada | Mental health counselling benefits | 2019 | - Indigenous considerations; - Mental healthcare service set-up/delivery |
| Government of Canada | Mental illness | 2020 | - Mental healthcare service Set-up/delivery; - Provincial/Territorial healthcare differences; |
| Government of Saskatchewan | Saskatchewan’s mental health and addictions action plan | 2019 | - Future directions of mental healthcare (what is being done to create improvements); - Mental healthcare service Set-up/delivery |
| Heal Action Lobby | End of 2004 health accord; federal leadership required. | 2014 | - Mental healthcare service Set-up/delivery; - Recommendations (improving federal government involvement with provincial/territorial and localized mental healthcare) |
| Health Canada | Canada health act annual report 2020-2021. | 2022 | - Barriers to mental healthcare (cost) - Mental healthcare service Set-up/delivery; - Provincial/Territorial healthcare differences; |
| Holloway, M., Holloway, G., & Witte, J. | Issues in emerging adulthood | 2010 | - Barriers (Cost, Socioeconomic status) |
| Howard, A. L., Galambos, N. L., & Krahn, H. J. | Paths to success in young adulthood from mental health and life transitions in emerging adulthood | 2010 | - Gender differences; - Transition of Emerging Adults |
| Johnston, S., & Hogel, M. | A decade lost: Primary healthcare performance reporting across Canada under the action plan for health system renewal | 2016 | - Healthcare system issues (delayed reporting; lack of commitment; lack of standardization); - Provincial/Territorial healthcare differences |
| Landberg, M., Lee, B., & Noack, P. | What alters the experience of emerging adulthood? How the experience of emerging adulthood differs according to socioeconomic status and critical life events | 2019 | - Socioeconomic status as impacting factor to mental health of Emerging Adults |
| MacLeod, K. B., & Brownlie, E. B. | Mental health and transitions from adolescence to emerging adulthood: Developmental and diversity considerations | 2014 | - Barriers (peer relations, Trauma, abuse, disadvantaged communities, socioeconomic status; wait times); - Diversity concerns; - Indigenous concerns; |
| McCloughen, A., Foster, K., Kerley, D., Delgado, C., & Turnell, A | Physical health and well-being: Experiences and perspectives of young adult mental health consumers | 2016 | - Barriers to mental healthcare (lack of mental health literacy; lack of faith in practitioners); - Importance of education; - Suggestions for improving mental healthcare (mental health literacy) |
| Mental Health Commission of Canada | Consensus conference on the mental health of emerging adults: Making transitions a priority in Canada | 2017 | - Barriers to mental healthcare (ageing out of services, long wait times) - Diversity concerns; - Indigenous considerations; - Mental healthcare service set-up/delivery; - Mental healthcare system concerns (lack of collaboration, ineffective system design) - Suggestions for improving mental healthcare (bridging service gaps, service changes, policies) |
| Mental Health Commission of Canada | Mental health supports for emerging adults. Where are they and what they do: A practices of interest directory | 2019 | - Barriers to mental healthcare (accessibility, ageing out of healthcare) - Mental healthcare service set-up/delivery; - Suggestions for improving mental healthcare (inclusion of emerging adults in policy/system creation) |
| Mental Health Commission of Canada | Mental health of emerging adults | 2022 | - Barriers to mental healthcare (ageing out of service) - Mental healthcare service set-up/delivery - Importance of early mental healthcare intervention |
| Moroz, N., Moroz, I., & Slovinec D’Angelo, M. | Mental health services in Canada: Barriers and cost-effective solutions to increase access. | 2020 | - Mental healthcare barriers (Personal circumstances, stigma, rural areas, long wait times, lack of preparedness in primary care physicians, lack of funding); - Suggestions to improve mental healthcare (community-based help, E-mental health/technology as support, funding allocation) |
| Mulvale, G., Roussakis, C., Canning, C., Papadodoulos, D., & Knoops, F. | Knowledge mobilization and mental health policy: Lessons from the Canadian consensus conference on the mental health of emerging adults | 2017 | - Recommendations for future healthcare policy; - Review of efficiency of consensus conference |
| Newcomb-Anjo, S. E., Barker, E. T., & Howard, A. L. | A person-centered analysis of risk factors that compromise wellbeing in emerging adulthood | 2016 | - Barriers (low socioeconomic status; social support; dispositional traits) |
| Taiminen, H., & Saraniemi, S. | Acceptance of online health services for self-help in the context of mental health: Understanding young adults’ experiences | 2018 | - Technology as a mental healthcare tool |
| Winzer, R., Lindblad, F., Sorjonen, K., & Lindberg, L. | Positive versus negative mental health in emerging adulthood: A national cross-sectional survey | 2014 | - Impacts of social supports |
